# EURISCO update 2023: the European Search Catalogue for Plant Genetic Resources, a pillar for documentation of genebank material

**DOI:** 10.1093/nar/gkac852

**Published:** 2022-10-03

**Authors:** Pragna Kotni, Theo van Hintum, Lorenzo Maggioni, Markus Oppermann, Stephan Weise

**Affiliations:** Leibniz Institute of Plant Genetics and Crop Plant Research (IPK) Gatersleben, Corrensstr. 3, 06466 Seeland, Germany; Centre for Genetic Resources, The Netherlands (CGN), Wageningen University & Research, Droevendaalsesteeg 1, 6708 PB Wageningen, The Netherlands; European Cooperative Programme for Plant Genetic Resources (ECPGR), c/o Alliance of Bioversity International and CIAT, Via di San Domenico 1, 00153 Rome, Italy; Leibniz Institute of Plant Genetics and Crop Plant Research (IPK) Gatersleben, Corrensstr. 3, 06466 Seeland, Germany; Leibniz Institute of Plant Genetics and Crop Plant Research (IPK) Gatersleben, Corrensstr. 3, 06466 Seeland, Germany

## Abstract

The European Search Catalogue for Plant Genetic Resources (EURISCO) is a central entry point for information on crop plant germplasm accessions from institutions in Europe and beyond. In total, it provides data on more than two million accessions, making an important contribution to unlocking the vast genetic diversity that lies deposited in >400 germplasm collections in 43 countries. EURISCO serves as the reference system for the Plant Genetic Resources Strategy for Europe and represents a significant approach for documenting and making available the world’s agrobiological diversity. EURISCO is well established as a resource in this field and forms the basis for a wide range of research projects. In this paper, we present current developments of EURISCO, which is accessible at http://eurisco.ecpgr.org.

## INTRODUCTION

Crop plants are the basis of nutrition for humans and farmed animals. However, biodiversity, including the agricultural component, is threatened worldwide by various factors (1–4). The progressing climate crisis plays a special role here (5,6). In particular, rising temperatures, changing precipitation patterns and the increasing frequency of extreme weather events are leading to adverse effects, such as declining crop yields (7). The changes in the environment due to the climate crisis are also forcing farmers to grow other varieties or even other crops, with the danger of losing the old ones. Furthermore, crop related wild species are increasingly under threat of disappearance due to these environmental changes (8). In order to combat this genetic erosion and be able to rely on the genetic diversity of cultivated plants and their wild relatives in the future, this diversity must be preserved. Genebanks play an important role in long-term preservation efforts. There are about 1800 genebank collections of plant genetic resources for food and agriculture (PGRFA) worldwide, of which about 625 are in Europe (9). It is a truism that something can only be used if one has information about it. The best resource will not be exploited if it is not well documented, and without data, proper genebank management will not be possible (10). In other words, the better described PGRFA material is, the more valuable it is for potential users and the better it can be preserved. In addition to pure management data and information on the legal status of the material, it is therefore important to have information that allows users to select the most suitable material for breeding and research programmes, especially passport data (about identity and origin) and phenotypic characterisations (about traits) of the genebank material (11).

The European Search Catalogue for Plant Genetic Resources (EURISCO) is an international aggregated database that aims to provide a central entry point for information on the large genetic diversity harboured in the collaborating collections. Presently, it contains data on more than two million genebank samples, so-called accessions, which are preserved in >400 institutes in Europe and some neighbouring countries. EURISCO is maintained on behalf of the European Cooperative Programme for Plant Genetic Resources (ECPGR) and is based on a network of National Inventory Focal Points, one in each of 43 member countries. EURISCO has been available online since 2003 and from 2014 the Leibniz Institute of Plant Genetics and Crop Plant Research (IPK) Gatersleben, Germany, has been responsible for the operation and further development of the information system as well as the coordination of the EURISCO network of National Inventory Focal Points.

## SYSTEM OVERVIEW

The National Inventory Focal Points of the EURISCO member countries are responsible for the development and maintenance of the National Inventories. These comprise of data about the PGRFA collections maintained by local curators in these countries under *ex situ* conditions. The data management of these collections is in many cases supported by IT systems that allow data exchange with the National Inventory Focal Points, which in turn regularly update the data in EURISCO (12). The basis for the core of the data collection (passport data) is the Multi-Crop Passport Descriptors standard (MCPD) (13). MCPD is the globally recognised standard for the documentation and exchange of passport data on plant genetic resources and enables the acquisition of data following a well-defined and legally legitimised procedure. Additionally, a EURISCO-specific format is used for the exchange of phenotypic data. The procedures for exchanging data with the National Inventory Focal Points are well established, but are constantly being adapted and improved.

EURISCO runs on an Oracle relational database management system using its specific technologies like PL/SQL and Oracle Application Express. The database backend includes a staging area for pre-processing and data cleansing (64 tables) as well as performance-optimised database structures for the web frontend (48 tables). The >200 functions and procedures within 23 packages mainly provide functionalities for data quality assurance, but also for user-specific download options, visualisations and statistics, among others. A web frontend with a broad range of functions is available for the users of the system. The frontend is meant to be user-friendly for any type of the targeted users, which include plant breeders and researchers, but also farmers, policy makers and other scholars. The web frontend is developed with Oracle Application Express technology, version 21.

## UPDATED DATABASE CONTENT AND STATISTICS

Currently, more than two million PGRFA accessions are documented in EURISCO. These include major cereal grains such as wheat (205 088), barley (126 330) and maize (67 869), pulses such as beans (55 344), peas (38 914) and vetches (29 438), edible oilseed crops such as sunflower (7735) and linseed (20 900), tuber crops such as potatoes (15 139), accessions of the *Brassica* complex (35 308), but also a very large research collection of *Arabidopsis thaliana*, a model plant in life sciences (684 967) is included. In total, the accessions documented in EURISCO comprise 6737 genera and 45 175 species. 428 160 of the accessions are part of the Multilateral System of Access and Benefit Sharing (MLS) of the International Treaty on Plant Genetic Resources for Food and Agriculture (ITPGRFA), which means that they have been confirmed by the respective countries to be promptly available under standard and internationally agreed benefit sharing conditions. In addition, there are 2.6 million records of phenotypic data from more than 3800 experiments conducted on genebank material.

Since the first description of EURISCO in the NAR database issue 2017 (12), the volume of data has been expanding continuously. The number of data provider institutions rose from 376 to 402. The number of accessions documented in the system increased by >200 000 (13%), details of which are illustrated in Table 1. During the same period, the number of phenotypic data points increased by 2.2 million (500%). This shows that EURISCO is steadily gaining acceptance as a repository of such data. Moreover, data on plant genetic resources are not static. Rather, they are continuously curated and expanded. On average, between 30 and 40 National Inventory datasets are updated each year, either in part or in full. These updates include data of on average 350 000–400 000 accessions per year. Regular training workshops, organised with the support of ECPGR, are held to support the data providers, which aim, among other things, to raise awareness of various aspects of data quality and to successively improve it.

**Table 1.** PGRFA accessions of the EURISCO database grouped by genus and species. The ten most common genera are broken down in detail. The total number of accessions per genus is shown in the column ‘Accs. total’. In addition, the figures from the first description of EURISCO in the NAR database issue 2017 (12) and the percentage change since then are shown

Genus	Species	No. of accs.	Accs. total	Weise *et al.* (2017)	Increase
*Arabidopsis*	*thaliana*	684 967	**685 192**	669 587	2.33%
	others	225			
*Triticum*	*aestivum*	145 483	**205 088**	147 055	39.46%
(wheat)	*durum*	17 482			
	*turgidum*	15 084			
	*monococcum*	3624			
	*spelta*	3419			
	*dicoccum*	1187			
	*aethiopicum*	1048			
	others	17 761			
*Hordeum*	*vulgare*	116 283	**126 330**	105 289	19.98%
(barley)	*spontaneum*	6172			
	others	3875			
*Zea*	*mays*	67 743	**67 869**	61 932	9.59%
(maize)	others	126			
*Phaseolus*	*vulgaris*	50 211	**55 344**	49 774	11.19%
(garden bean)	*coccineus*	2329			
	others	2804			
*Solanum*	*lycopersicum*	22 429	**53 295**	44 400	20.03%
(tomato, potato, eggplant, etc.)	*tuberosum*	15 139			
	*andigenum*	2814			
	*melongena*	2219			
	others	10 694			
*Vitis*	*vinifera*	38 295	**47 488**	28 819	64.78%
(grape)	others	9193			
*Avena*	*sativa*	33 838	**42 405**	29 429	44.09%
(oat)	*sterilis*	2401			
	*byzantina*	1988			
	others	4178			
*Pisum*	*sativum*	37 424	**38 914**	29 735	30.87%
(pea)	others	1490			
*Malus*	*domestica*	32 553	**34 558**	27 698	24.77%
(apple)	others	2005			
others			**723 029**	649 034	11.40%
Total			**2 079 512**	1 842 752	12.85%

Stable and unique identifiers are an important prerequisite for the operation of aggregated databases such as EURISCO (10), allowing the information in EURISCO to be linked easily to information in other data sources, especially where it concerns genomics and other omics data, that are not included in EURISCO. Digital Object Identifiers (DOIs) have established themselves as a quasi-standard in the field of plant genetic resources in recent years (14). Therefore, the increasing acceptance of DOIs is a very positive development. In close cooperation with the Secretariat of the ITPGRFA, EURISCO offers its data providers the service of carrying out the DOI registration of their accessions on their behalf. This service is well established, as reflected in the continuous increase in the number of DOIs.

## ENHANCED WEB INTERFACE

**Figure 1.**
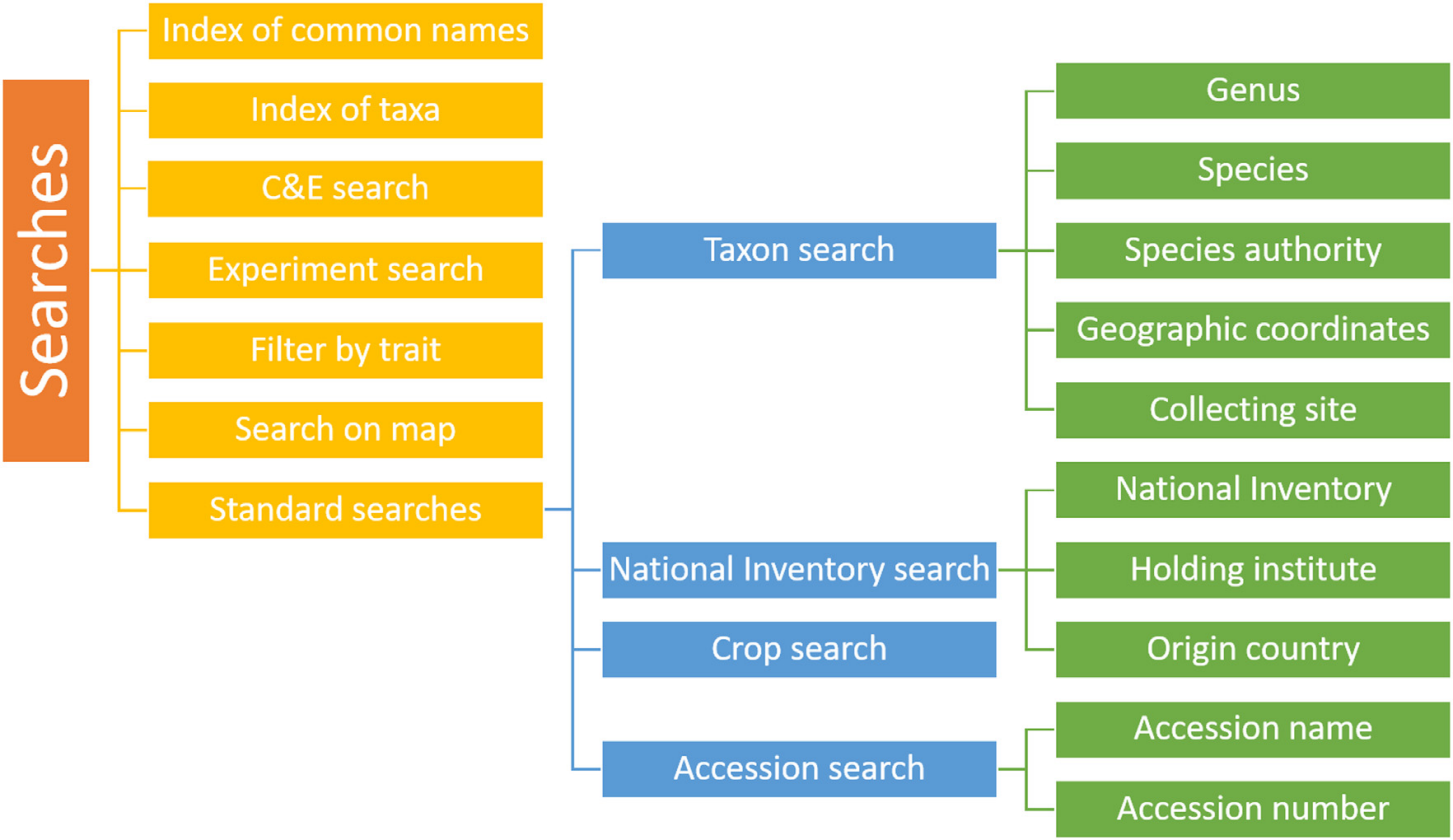
The different search capabilities of EURISCO.

**Figure 2.**
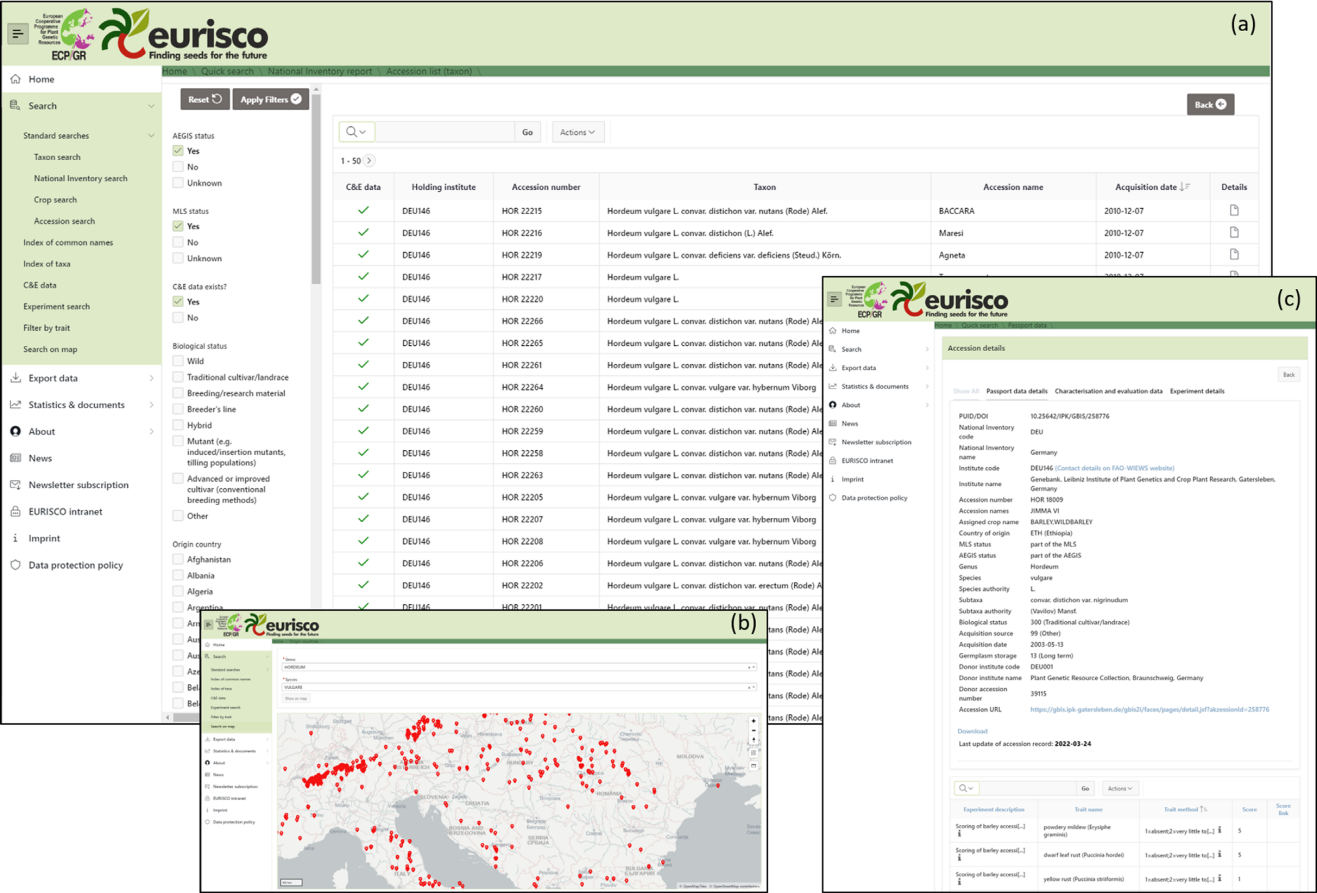
Overview of the EURISCO web interface. (**A**) The result set of a search query, which can be further narrowed down using facets. (**B**) A map-based search. (**C**) A detailed view of passport and phenotypic data of a selected accession.

EURISCO’s central user interface has recently been completely revised. It offers a variety of ways to find information (Figure 1). In addition to various wizard-based standard searches, indexes of common crop names and taxonomic terms are offered to provide searches at different levels. Map-based searches are also possible. The results can then be narrowed down using further filters, e.g. faceted searches (Figure 2). Various data export features allow data to be downloaded for later use. In addition to passport data searches, wizards offer the possibility to search for phenotypic data.

One feature that should be highlighted is the sophisticated taxonomic search in EURISCO. When searching by taxonomic names, users often face the challenge that different PGRFA collections follow different taxonomic schools and traditions, resulting in a variety of accepted names and synonyms. Therefore, a feature was implemented for EURISCO that automatically maps the scientific names of all accessions against different taxonomic repositories representing different taxonomic opinions. This leads to much more complete result sets. Currently, the mapping is done against GRIN Taxonomy (15) as well as the Mansfeld’s World Database of Agricultural and Horticultural Crops, which is based on the Mansfeld’s Encyclopedia of Agricultural and Horticultural Crops (16). The taxonomic search feature was first described in (17) and has been continuously expanded since then.

Another feature is the uniform search for common crop names. Thus far, there is no globally accepted controlled vocabulary for this. As a result, the PGRFA accessions in EURISCO are described with arbitrary crop names or no crop name at all, which makes a uniform search for accessions of a specific crop across all accessions impossible. For this reason, a mechanism was developed that automatically maps all accessions in EURISCO against the crop names used in GRIN (15), regardless of what data, if any, were provided by the National Inventories for ‘crop name’. This makes it possible to search uniformly across all two million accessions and to achieve significantly better matching results.

## SUMMARY AND PERSPECTIVES

EURISCO’s main task is to provide a central entry point for information on plant genetic resources maintained *ex situ* in participating institutions. This presents an important contribution to opening up a large part of the millions of accessions preserved in germplasm collections worldwide for research and breeding. In particular, old landraces and crop wild relatives documented in EURISCO can provide important contributions to future breeding efforts. In this context, it should not go unmentioned that an extension of EURISCO to include data on *in situ* crop wild relatives’ populations is currently under preparation and will further strengthen the role of the system.

In addition, work is ongoing to expand EURISCO’s role as a repository of phenotypic data on PGRFA accessions. The amount of corresponding data has increased significantly in recent years. Compliance with the criteria defined by FAIR (18) is an important goal for today’s research data management. However, the phenotypic data managed in EURISCO cannot fully comply with these criteria. In the genebank community, a major challenge is that there is a large variety of small, relatively poorly described phenotyping datasets. Some of these are several decades old, but represent important sources for the use of PGRFA material and are indispensable against this background. Due to the history of the data, traits and methods used are often heterogeneous. Thus, an important goal is to achieve full compliance with MIAPPE (Minimum Information about Plant Phenotyping Experiments (19)) in order to improve the FAIRness of the data. To further improve access to the data available in EURISCO, the implementation of Breeding API (BrAPI) (20) endpoints is being evaluated. Moreover, the various research projects EURISCO is involved in are used to develop and test further extensions, such as an improved linkage with genetic data. The web interface will continue to be developed in the coming years.

EURISCO is also the European node of the international PGRFA information system Genesys, operated by the Global Crop Diversity Trust, and provides the passport data of the accessions maintained *ex situ*. The phenotypic data mentioned above are provided exclusively through EURISCO. The same applies to the *in situ* crop wild relative data that will soon be included in EURISCO. Together with systems like Genesys or GRIN-Global, EURISCO forms a vital component of the global information system (GLIS) (21). It is expected that EURISCO will remain an important resource in the field of plant genetic resources management and use, and will continue to form the basis for a wide range of research projects. Its further strengthening and development has been included among the 2030 targets of the Plant Genetic Resources Strategy for Europe (22).

## DATA AVAILABILITY

EURISCO will be continuously maintained and updated, and is publicly accessible at http://eurisco.ecpgr.org. After its launch in 2003 as a product of an EU-funded project, continuity of funding of EURISCO has been guaranteed in the past twenty years with contributions mainly from the European countries that are members of ECPGR.
